# Supplementary material for: Viral detection and identification in 20 min by rapid single-particle fluorescence in-situ hybridization of viral RNA
Source: Sci Rep. 2021 Oct 1;11:19579. doi: 10.1038/s41598-021-98972-z (PMC8486776; doi:10.1038/s41598-021-98972-z)
Supplement: Supplementary file 1 — Supplementary Information. [file 41598_2021_98972_MOESM1_ESM.docx]

Table S1: WSN FISH probe set

| **probe #** | **sequence** |
| --- | --- |
| 1 | gttcaccattgacaagtagt |
| 2 | agattggtcttggccagacg |
| 3 | gtggtgtgaatagtgatact |
| 4 | agtgggagcatcatttcttt |
| 5 | tgaggaggacgcaatctgga |
| 6 | ttgaattaatcagggggcta |
| 7 | tgtatgaggccttgcttctg |
| 8 | tcctgagctaacagggctag |
| 9 | acagcggaagtttcgttcaa |
| 10 | gtggcaataactaatcggtc |
| 11 | agtaggttctctatgagaca |
| 12 | cctaatggatggacagagac |
| 13 | tgggtttgagatgatttggg |
| 14 | ggactaaaagtgacagttcc |
| 15 | agtatggcaatggtgtttgg |
| 16 | cggagtaaagggattttcat |
| 17 | cagtgtctgctgatggagca |
| 18 | ccaaagatggaacaggcagc |
| 19 | taggatacatctgcagtggg |
| 20 | ccttcgaccaaaacctagat |
| 21 | cacggttcgaaccgaccatg |
| 22 | gtgtgtgtgcagagacaatt |
| 23 | ttaccctgataccggcaaag |
| 24 | ctcactacgaggaatgttcc |
| 25 | caatagagttgaatgcacct |
| 26 | tcgagaaggggaaggttact |
| 27 | gctggcctcgtacaaaattt |
| 28 | ttaccataatgaccgatggc |
| 29 | gagtctgaatgtacctgtgt |
| 30 | ccgcataataactgaaacca |
| 31 | ggagcagtggctgtattaaa |
| 32 | acaatcggaatttctggtcc |
| 33 | agtgcatgtcatgatggagt |
| 34 | tgaatcggttgcttggtcag |
| 35 | cgtccccgtacaattcaagg |
| 36 | ttatagggccttaatgagct |
| 37 | ggggacctttaaggacagaa |
| 38 | cgccttactgaatgacaagc |
| 39 | ggaccttttttctgactcaa |
| 40 | atttcatgttctcacttgga |
| 41 | gtttttgtcataagagagcc |
| 42 | tggcataagaattggttcca |
| 43 | gtgggctatacacagcaaag |
| 44 | gaatatgcaaccaaggcagc |
| 45 | agccattcaattcaaaccgg |
| 46 | ggtagtcggaataattagcc |
| 47 | taaccattgggtcaatctgt |
| 48 | gcaggagtttaaatgaatcc |

Table S2: IBV FISH probe set

| probe # | sequence |
| --- | --- |
| 1 | ttatgacagtgggcatttct |
| 2 | aaaactgccttttctacacc |
| 3 | ccacagatgtaggtagagtt |
| 4 | ttctctttgcatatagctca |
| 5 | aatacggttgtttggcagtg |
| 6 | cgtctacacctaaacctttc |
| 7 | tcccaaattacaaatccatt |
| 8 | ccttgacggtattacgatac |
| 9 | agaactactaggccatttgg |
| 10 | atcaccatatctatcatcat |
| 11 | cgcttataacactgtgtaga |
| 12 | ggcataccattctgaacgag |
| 13 | taaacatacaggttcgctcc |
| 14 | aaacgcaccattaacacgct |
| 15 | tttattgtgttaggtagtgt |
| 16 | tttcataacttcgaccctgg |
| 17 | atgtcactacgaggttcaaa |
| 18 | catagcgagaaaatcacgct |
| 19 | tttctacaaaactctcctct |
| 20 | aggcctaagtctttaccata |
| 21 | ttcaccatacagtatgtgtt |
| 22 | cacctaattggggcttatca |
| 23 | acatacctataacagtgtgt |
| 24 | ttattcgcacgtaagagtct |
| 25 | agttacagactttgcgttca |
| 26 | tgcatgacatcagaatccga |
| 27 | gtaagaaccattgtccgaca |
| 28 | ccacaacagtacacacttgc |
| 29 | agtaccatactccttaagta |
| 30 | cactgttacaacttttgact |
| 31 | actgccatcttcaaaccaag |
| 32 | agctgtggataacatgtttt |

Table S3: SARS-Cov-2 FISH probe set

| probe # | sequence |
| --- | --- |
| 1 | aacgattgtgcatcagctga |
| 2 | gtcaaaagccctgtatacga |
| 3 | accagctactttatcattgt |
| 4 | aattgtcatcttcgtccttt |
| 5 | catgtttagcaacagctgga |
| 6 | catgtcaccgtctattctaa |
| 7 | tctgccattgtgtatttagt |
| 8 | ttcacctaagttggcgtata |
| 9 | cgcatggcatcacagaattg |
| 10 | gtacaccaacaataccagca |
| 11 | ccctggtcaaggttaatata |
| 12 | caacatgtgactctgcagtt |
| 13 | cccacttaatgtaaggcttt |
| 14 | tctcttccgtgaagtcatat |
| 15 | atttgggtggtatgtctgat |
| 16 | tgcagaatgcatctgtcatc |
| 17 | gtggtccaaaacttgtaggt |
| 18 | caacacctagctctctgaag |
| 19 | tagattaccagaagcagcgt |
| 20 | acgtagtgcgtttatctagt |
| 21 | agaaacccttagacacagca |
| 22 | tgacgatgacttggttagca |
| 23 | aaccagctgatttgtctagg |
| 24 | agatagagacaccagctacg |
| 25 | tggcggctattgatttcaat |
| 26 | ccatagaatttgcttgttcc |
| 27 | gtgagggttttctacatcac |
| 28 | cggtgtgacaagctacaaca |
| 29 | acttgagcacactcattagc |
| 30 | acacatgaccatttcactca |
| 31 | aagtgcattaacattggccg |
| 32 | cggcaattttgttaccatca |
| 33 | cacaacagcatcgtcagaga |
| 34 | accttgagatgcataagtgc |
| 35 | aagttctttatgctagccac |
| 36 | tcagtccaacattttgcttc |
| 37 | tgaggtcctttagtaaggtc |
| 38 | tggatctgggtaaggaaggt |
| 39 | gcatactcctgattaggatg |
| 40 | cccaataccttgaagtgtta |
| 41 | aagactgtatgcggtgtgta |
